# False-positive asynchronous-abdominal curtain sign mimicking pneumothorax in cats with severe pulmonary hyperinflation—case report

**DOI:** 10.3389/fvets.2026.1707703

**Published:** 2026-01-27

**Authors:** Pablo A. Donati, Gustavo A. Plotnikow, Søren R. Boysen

**Affiliations:** 1 Cátedra de Anestesiología y Algiología, Facultad de Ciencias Veterinarias, Universidad de Buenos Aires, Buenos Aires, Argentina; 2 Cooperative Veterinary Intensive Care Unit (UCICOOP), Buenos Aires, Argentina; 3 Servicio de Rehabilitación, Unidad de Terapia Intensiva, Hospital Británico de Buenos Aires, Buenos Aires, Argentina; 4 Facultad de Medicina y Ciencias de la Salud, Universidad Abierta Interamericana, Buenos Aires, Argentina; 5 Department of Veterinary Clinical and Diagnostic Sciences, Faculty of Veterinary Medicine, University of Calgary, Calgary, AB, Canada

**Keywords:** anaphylaxis, curtain sign, feline, feline lower airway disease, POCUS

## Abstract

**Introduction:**

The asynchronous-abdominal curtain sign (A-ACS) is a suggestive ultrasound artifact for pneumothorax in veterinary medicine. However, its specificity has not been fully established.

**Case presentation:**

We describe two dyspneic cats displaying a prominent A-ACS despite the absence of findings strongly supportive of pneumothorax. Both cases had severe pulmonary hyperinflation secondary to expiratory flow limitation (feline lower airway disease and suspected anaphylaxis). Sonographic assessment revealed A-ACS and reduced diaphragmatic excursion. Medical management targeting bronchoconstriction and inflammation resulted in complete resolution of respiratory signs, normalization of the abdominal curtain sign (ACS), and improved diaphragmatic excursion in the monitored case.

**Discussion:**

These findings suggest that severe pulmonary hyperinflation may represent a cause of false-positive A-ACS, likely resulting from diaphragmatic flattening and altered cardiophrenic mechanics.

**Conclusion:**

Clinicians should consider pulmonary hyperinflation in cats presenting with A-ACS to avoid misdiagnosis of pneumothorax and unnecessary interventions. Moreover, this sign may offer valuable insights into respiratory dynamics in feline obstructive airway disease.

## Introduction

In point-of-care lung ultrasound, the asynchronous-abdominal curtain sign (A-ACS) has emerged as a dynamic artifact suggestive of pneumothorax in dogs and cats (1, 2). It is characterized by a dyssynchronous movement of the air–tissue interface (i.e., the interface between aerated lung and adjacent abdominal soft tissues), opposite to abdominal motion, classically interpreted as reflecting dissociation of the lung from the thoracic wall by free pleural air (3). Operationally, the A-ACS is identified when caudal abdominal structures and diaphragmatic motion move cranially during inspiration without a corresponding synchronous movement of the visceral pleural line or adjacent lung surface, resulting in a visible temporal dissociation between abdominal and pleural motion. This sign addresses the limitations of relying solely on the absence of lung sliding, which can yield false positives in patients with rapid shallow breathing (4).

However, the diagnostic performance of the A-ACS has not been formally established in veterinary patients. We hypothesize that pathophysiologic conditions associated with marked alterations in lung–thoracic wall mechanical interactions, without true pleural separation, could produce a false-positive A-ACS. Severe pulmonary hyperinflation, a common consequence of expiratory flow limitation in feline lower airway disease (FLAD), provides a plausible pathophysiologic basis for this phenomenon (5, 6). The associated diaphragmatic flattening and functional impairment reduce diaphragmatic excursion and alter normal lung–thoracic wall mechanics (7). In this manuscript, pulmonary hyperinflation is defined as a functional radiographic and physiologic state characterized by increased lung volume and diaphragmatic flattening secondary to expiratory flow limitation, rather than as a primary structural lung disease.

This report describes two cats with pulmonary hyperinflation due to expiratory flow limitation, in which an A-ACS was observed despite the absence of pneumothorax. We aim to alert clinicians that severe pulmonary hyperinflation is a critical differential diagnosis for a positive A-ACS. Recognizing this mimic is essential to prevent misdiagnosis of pneumothorax and avoid unnecessary and potentially iatrogenic interventions such as thoracocentesis.

## Case description

During ultrasound examinations, cats were maintained in sternal recumbency. A portable ultrasound unit (Sonoscape S6, SonoScape Medical Corp., Shenzhen, China) equipped with a microconvex transducer (4–8 MHz) was used for all assessments. Fur was not clipped, and alcohol was used as a coupling agent. Lung ultrasound was performed using a thoracic vertical sweeping technique, with the transducer placed longitudinally in the intercostal spaces and slid horizontally in a caudal direction, one intercostal space at a time, until the abdominal curtain sign (ACS) was identified. Diaphragmatic excursion was assessed from a subxiphoid view using M-mode, with the probe oriented cranially to visualize diaphragmatic motion during spontaneous respiration. Ultrasound settings (depth and gain) were adjusted according to the target structure, with different presets used for the evaluation of A-ACS and diaphragmatic excursion, respectively.

Case 1: A 10-year-old cat was referred for worsening dyspnea. The patient had a history of FLAD of 10 years’ duration and had been treated with inhaled fluticasone and salmeterol. At presentation, a thoracic vertical sweeping ultrasound protocol was performed (7), revealing A-lines and preserved lung sliding. No focal absence of lung sliding was identified in the regions evaluated, including at the level of the ACS. The evaluation of the ACS showed asynchronous movement, and the diaphragmatic excursion appeared reduced. Given the concern for pneumothorax raised by this finding, a thoracocentesis was immediately performed; this yielded no air and, when interpreted alongside the overall ultrasonographic assessment and subsequent thoracic radiographs, supported a low likelihood of pneumothorax rather than definitive exclusion. Subsequent thoracic radiographs demonstrated increased lung volume, increased pulmonary radiolucency, and diaphragmatic flattening, consistent with pulmonary hyperinflation. Dexamethasone (0.4 mg/kg) (DEXA 20, Richmond Vet Pharma, Buenos Aires, Argentina) was administered, and hospitalization was recommended, but the owner declined, and follow-up was lost.

Case 2: An 8-year-old cat was referred for dyspnea of 12 h’ duration. Initial laboratory evaluation revealed elevated urea and creatinine. The referring veterinarian administered subcutaneous fluids and vitamin B complex. Seventy-two hours later, fluids, vitamin B complex, and maropitant were repeated. Upon returning home, the cat developed marked dyspnea. Approximately 18 h later, it was taken to a veterinary clinic, hospitalized, and referred to a tertiary care center.

**Figure 1:**
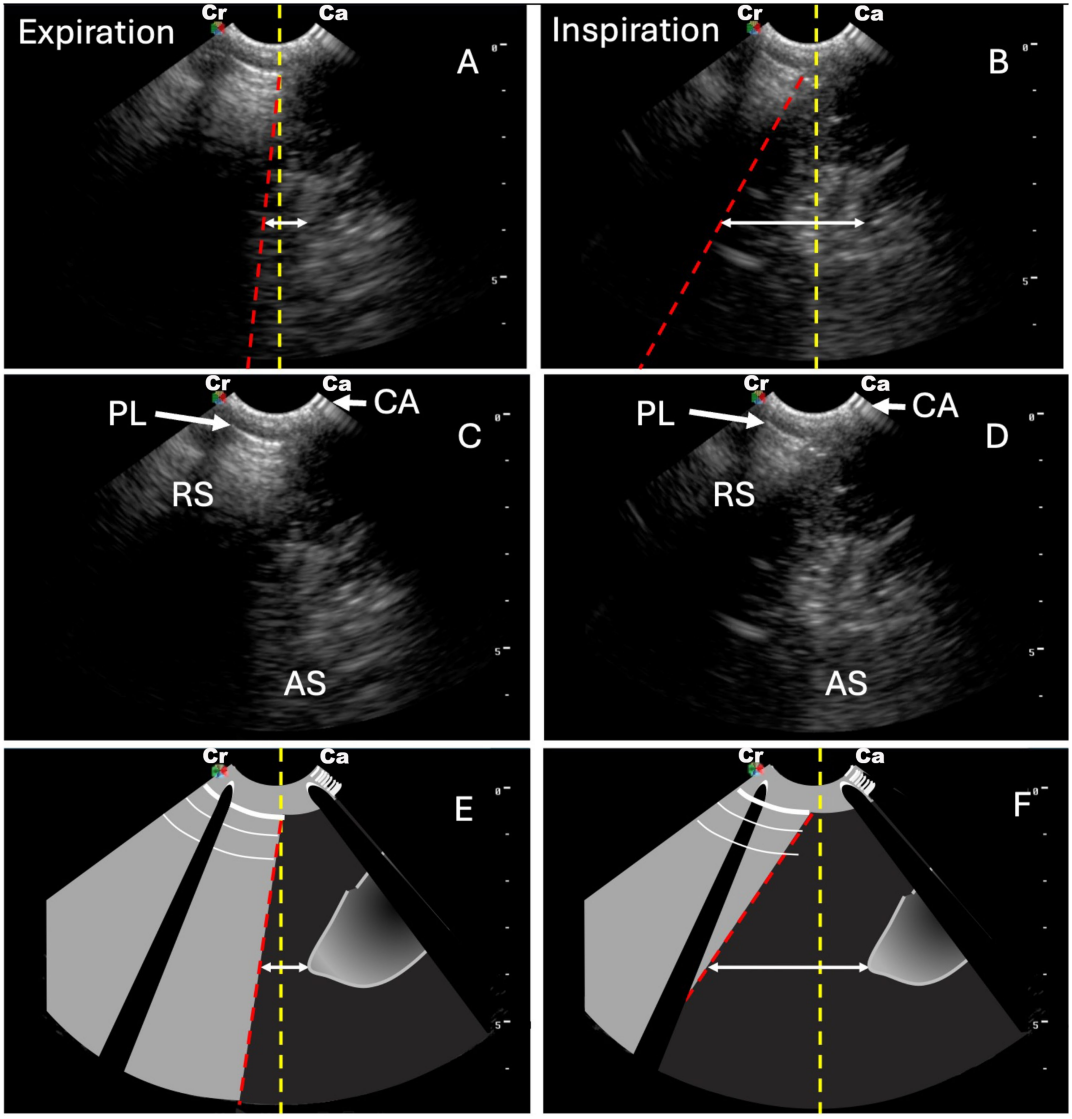
Still sonograms **(A–D)** and schematic images **(E–F)** of the asynchronous-abdominal curtain sign (A-ACS) obtained from Case 2 during expiration and inspiration. **(A,B)** Still sonograms with the abdominal curtain sign (ACS) (red dotted line) and a vertical reference line where the ACS originates from the pleural line during expiration (yellow dotted line). The white double-headed arrow shows the distance between the ACS and a specific abdominal structure. During expiration, the ACS and the abdominal structures move closer to each other **(A)**, and during inspiration, they move away from each other **(B)**. **(C,D)** Labelled still sonograms of **A** and **B** during expiration **(C)** and inspiration **(D)**. **(E,F)** Schematic drawings of the still sonograms shown in **A–D** during expiration **(E)** and inspiration **(F)**. RS, rib shadow; PL, pleural line; CA, contact artifact; AS, abdominal structures.

**Figure 2:**
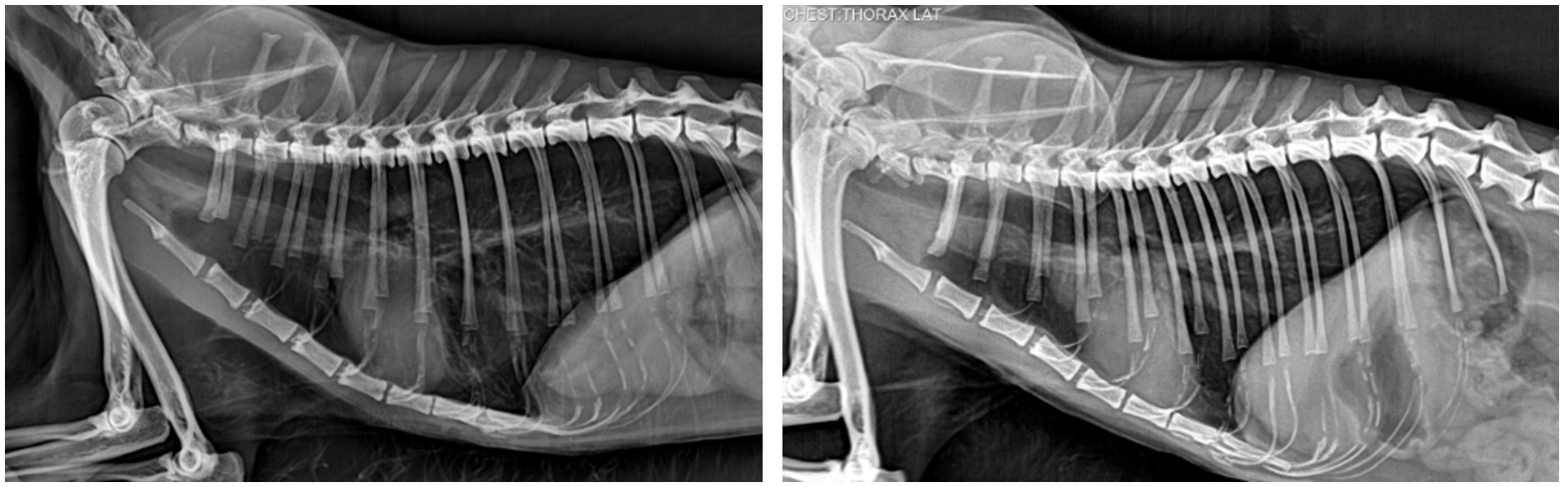
Radiographic features of pulmonary hyperinflation in a cat. Lateral thoracic radiographs show severe pulmonary hyperinflation characterized by increased thoracic volume, caudal displacement, and flattening of the diaphragm, and diffusely increased pulmonary radiolucency consistent with air trapping (left), with marked radiographic improvement after treatment (right).

On admission, the cat presented with dyspnea characterized by marked expiratory effort and concurrent inspiratory difficulty. A thoracic vertical sweeping ultrasound protocol was performed (8), revealing A-lines and preserved lung sliding. Additionally, an A-ACS (Supplementary material 1; Figure 1) and reduced diaphragmatic excursion (0.23 cm) were noted. Thoracic radiographs demonstrated increased lung volume, increased pulmonary radiolucency, and diaphragmatic flattening, consistent with pulmonary hyperinflation (Figure 2).

Due to the acute onset and absence of prior respiratory signs, anaphylaxis, possibly related to vitamin B administration, was suspected. Dexamethasone (0.4 mg/kg) (DEXA 20, Richmond Vet Pharma, Buenos Aires, Argentina) was administered, and nebulized salbutamol and ipratropium (Combivent SM, Boehringer Ingelheim, Ingelheim am Rhein, Germany) were initiated hourly, then every 2 h, until a marked improvement in respiratory pattern was observed. Follow-up ultrasound 24 h after admission revealed complete resolution of the A-ACS, now appearing synchronous (Supplementary material 2), alongside a significant improvement in diaphragmatic excursion (0.70 cm). This resolution correlated with a marked radiographic improvement of hyperinflation-related findings (Figure 1). The patient was discharged 48 h after admission.

## Discussion

This report describes two cases of cats presenting with acute dyspnea and expiratory flow limitation, who exhibited an A-ACS on thoracic ultrasound despite the absence of pneumothorax. While the A-ACS has been proposed as a sonographic sign suggestive of pneumothorax, the present cases suggest that false positives may occur, particularly in hyperinflated lungs without definitive sonographic evidence of pneumothorax. This underscores the critical importance of interpreting point-of-care lung ultrasound findings within the full clinical context.

In humans, obstructive airway disease is strongly associated with pulmonary hyperinflation and diaphragmatic dysfunction, with reduced diaphragmatic excursion commonly reported (7). In cats with expiratory flow limitations, hyperinflation is a relatively common feature that can lead to increased alveolar dead space, hypoxemia, and dyspnea (5). Anaphylactic reactions may also present with a similar asthma-like clinical picture, as the lung is considered the primary shock organ in cats (9). The specific effect of hyperinflation on diaphragmatic function has been less well characterized in feline patients. A recent study compared different sonographic markers of diaphragmatic function, including diaphragmatic excursion, and reported significant differences between healthy cats and those with pulmonary parenchymal abnormalities, pleural effusion, and cardiovascular disease (10), suggesting it is a relevant metric. In Case 2, a marked improvement in diaphragmatic excursion was documented following resolution of hyperinflation, suggesting that the impaired excursion was most likely secondary to hyperinflation rather than intrinsic diaphragmatic pathology, supporting the concept that diaphragmatic impairment in cats can be functional and reversible.

Pulmonary emphysema was considered as a differential diagnosis. However, emphysema is considered uncommon in cats and is typically associated with structural and often irreversible alveolar abnormalities. In the present cases, the rapid and near-complete clinical, sonographic, and radiographic improvement following medical therapy strongly supports functional pulmonary hyperinflation secondary to expiratory flow limitation rather than primary emphysematous disease.

Other conditions that may theoretically alter pleural motion or regional lung–thoracic wall coupling and potentially influence the appearance of the ACS were also considered. These include pleural adhesions, severe parenchymal lung disease, and fibrosing processes, all of which could reduce regional lung motion without true pleural separation. However, these differentials were considered less likely in the present cases due to the absence of sonographic or radiographic evidence of pleural thickening, pleural irregularity, or diffuse fibrotic lung disease, as well as the rapid reversibility of the sonographic findings following medical management in Case 2.

Although speculative, the pathophysiological basis for the A-ACS in these hyperinflated cats remains hypothetical and may be explained by the profound mechanical dysfunction of the diaphragm and the consequent compensatory mechanisms described in human obstructive lung disease. Chronic hyperinflation places the diaphragm at a severe mechanical disadvantage, causing chronic shortening, flattening, and a caudal displacement within the thoracic cavity (11). This compromised position drastically reduces diaphragmatic excursion and diminishes the zone of apposition, thereby impairing the ability of the diaphragm to descend effectively and expand the lower rib cage synchronously with abdominal content movement. To compensate, ventilation becomes increasingly dependent on rib cage inspiratory muscles (12). This mechanical uncoupling does not imply pleural separation, but rather an alteration in the normal synchrony between diaphragmatic motion, abdominal displacement, and lung expansion.

We hypothesize that this shift in the primary work of breathing to accessory muscles, combined with a weakened and poorly coupled diaphragm, creates regional disparities in intrathoracic pressure and airflow. This could theoretically lead to asynchronous movement of lung segments at the costophrenic recess (a phenomenon akin to pendelluft) (13), which manifests sonographically as an abnormal, A-ACS despite the presence of intact pleural sliding and the apparent absence of pneumothorax. Although not specifically noted in either of the cats in the current case series, it is possible that paradoxical breathing, which has been reported in cases of FLAD (14, 15), could also contribute to asynchrony between the ACS and abdominal structures.

A counterargument is the fact that FLAD has been associated with spontaneous pneumothorax, and that ultrasound might detect a radiographically occult pneumothorax (16, 17). However, pneumothorax was considered unlikely based on the overall clinical evolution, lack of air retrieval during thoracocentesis in Case 1, and the resolution of the A-ACS with medical management alone in Case 2. Nevertheless, the presence of a small or loculated pneumothorax cannot be absolutely excluded. Computed tomography (CT), considering the reference standard for diagnosis of pneumothorax, may have provided further evidence against this argument.

Although the presence of pleural lung slide is useful for ruling out pneumothorax, its absence—traditionally regarded as a supportive sonographic sign—must be interpreted with caution in small animal patients, as it may lead to false-positive suspicion of pneumothorax. Poor agreement between TFAST and CT for pneumothorax detection in dogs and cats has been reported, highlighting the limitations of relying on individual sonographic signs in isolation. Similarly, the present case suggests that an A-ACS may also be observed in the absence of pneumothorax, particularly in cats with severe pulmonary hyperinflation. Therefore, these findings should be interpreted in conjunction with other sonographic features and the overall clinical context.

Thoracocentesis carries a recognized risk of iatrogenic pneumothorax, particularly in patients with hyperinflated lungs. This possibility was considered in the present cases. However, no post-procedural clinical deterioration, sonographic evidence of pneumothorax, or radiographic worsening was observed. In Case 2, thoracocentesis was not performed, and the A-ACS resolved completely with medical management alone, further supporting a non-pneumothorax etiology.

Based on the findings in the 2 cases of this report, cats that have an A-ACS sonographically identified should have a history, signalment, and other clinical and sonographic diagnostic criteria assessed to support or refute a diagnosis of pneumothorax. The double curtain sign, which has been documented in dogs but not in cats, may be a more specific sonographic sign of pneumothorax than the A-ACS (1). The absence of lung sliding (also known as the glide sign) can be used to support a diagnosis of pneumothorax, but should be viewed with caution as it can be diminished in human patients with asthma (18), which may also be the case with FLAD. Finally, the lung point can be sought in patients sufficiently stable to tolerate longer sonographic examinations, although the sensitivity and specificity of this finding in cats and dogs with pneumothorax have yet to be determined in the clinical setting (15). Additional imaging modalities, including radiographs and CT, should also be considered if patients are sufficiently stable to allow further imaging and the diagnosis is uncertain.

This report has several limitations inherent to its retrospective, descriptive nature and small sample size. Orthogonal thoracic radiographic projections were not available at all time points, which may have limited the full radiographic characterization of pulmonary changes. Additionally, while pneumothorax was considered unlikely based on clinical evolution, imaging findings, and thoracocentesis results, the presence of a small or loculated pneumothorax cannot be absolutely excluded, particularly in the absence of CT. Finally, resolution of the A-ACS sign with medical management alone cannot definitively rule out spontaneous resolution of a small pneumothorax, particularly in the absence of CT.

In conclusion, these findings highlight the importance of integrating lung ultrasound signs with knowledge of respiratory mechanics and underlying pathophysiology. Recognition of an A-ACS in hyperinflated patients may help avoid misdiagnosis and unnecessary interventions. When an A-ACS is identified—particularly in a dyspneic cat with marked expiratory effort—clinicians should prioritize the search for evidence of pulmonary hyperinflation, including thoracic radiography and reduced diaphragmatic excursion, before considering invasive procedures such as thoracocentesis. Moreover, it provides a novel sonographic window into respiratory muscle function and regional ventilation in feline lower airway disease. Further characterization of the A-ACS may improve ultrasound accuracy and deepen our understanding of feline respiratory physiology.

## Data Availability

The original contributions presented in the study are included in the article/Supplementary material, further inquiries can be directed to the corresponding author.
